# Beyond Measurement and Reward: Methods of Motivating Quality Improvement and Accountability

**DOI:** 10.1111/1475-6773.12413

**Published:** 2015-11-10

**Authors:** Robert A. Berenson, Thomas Rice

**Affiliations:** ^1^ Health Policy Center The Urban Institute Washington DC; ^2^ Department of Health Policy and Management UCLA Fielding School of Public Health Los Angeles CA

**Keywords:** Physician payment, quality improvement, financial incentives, nonfinancial incentives, value‐based purchasing, pay‐for‐performance

## Abstract

**Objective:**

The article examines public policies designed to improve quality and accountability that do not rely on financial incentives and public reporting of provider performance.

**Principal Findings:**

Payment policy should help temper the current “more is better” attitude of physicians and provider organizations. Incentive neutrality would better support health professionals’ intrinsic motivation to act in their patients’ best interests to improve overall quality than would pay‐for‐performance plans targeted to specific areas of clinical care. Public policy can support clinicians’ intrinsic motivation through approaches that support systematic feedback to clinicians and provide concrete opportunities to collaborate to improve care. Some programs administered by the Centers for Medicare & Medicaid Services, including Partnership for Patients and Conditions of Participation, deserve more attention; they represent available, but largely ignored, approaches to support providers to improve quality and protect beneficiaries against substandard care.

**Conclusions:**

Public policies related to quality improvement should focus more on methods of enhancing professional intrinsic motivation, while recognizing the potential role of organizations to actively promote and facilitate that motivation. Actually achieving improvement, however, will require a reexamination of the role played by financial incentives embedded in payments and the unrealistic expectations placed on marginal incentives in pay‐for‐performance schemes.

An overarching goal of health care policy makers in both the public and private sectors is to provide an environment where health care providers can produce safe, high‐value patient care. While there are numerous ways to work toward achieving this, policy makers and payers have focused mostly on coupling financial incentives with performance measurement. The two most common methods are (1) pay‐for‐performance (P4P) programs that explicitly tie provider payments to the achievement of particular results, and (2) public reporting, usually in the form of health care report cards that rate, compare, and make available to the public measures of provider performance.

In this article, we argue that mechanisms to promote improved quality and value using approaches other than financial incentives and performance measurement, while heretofore largely neglected in public policy, have the potential to be especially effective in fostering society's goals and may result in fewer undesirable side effects. Depending on the circumstances, these alternative approaches may either replace or complement financial incentives and performance measurement. This article applies most directly to physicians and hospitals, but the arguments can be applied to other individuals and organizations in the health sector as well.

We begin by providing a brief discussion of some understudied aspects of financial incentives to inform the discussion of public policies that follows. This includes background on implicit incentives, which mainly rely on trust rather than financial inducements or regulation; incentive neutrality, which aims to prompt providers to act in the best interest of patients by providing high‐quality care and respecting their wishes and, at the same time, being mindful of societal interests in preserving scarce resources; and marginal versus embedded incentives, arguing that the latter may be more important in motivating providers than the typical explicit incentives inherent in P4P systems.

The second section begins by reviewing recent findings demonstrating the limited role of financial incentives in improving surgical care and suggesting that efforts that do not rely primarily on financial incentives likely would be more effective. The third section explores a range of approaches to improving quality and promoting accountability, and discusses ways in which payers can motivate individuals and organizations to foster quality improvement (raise the mean of performance) without relying primarily on financial incentives.

## Some Understudied Aspects of Financial Incentives

Financial incentives are the primary mechanism for motivating behavior in a marketplace, whether they are aimed at consumers or suppliers. The dominance of such factors in economic analysis is illustrated by the fact that microeconomic theory has just one independent variable—price—on the standard supply and demand graph. Yet a myriad of factors besides price affect the use of care, just as many nonfinancial incentives affect the provision of quality care.

## Implicit Incentives

A simple typology of incentives in medical practice is shown in Table 1 (Maynard 2006). Incentives are divided between implicit and explicit, and internal and external. The implicit incentives are based on trust: doing the best one can for the patient (internal) and earning the trust of consumers and other providers (external). Explicit incentives include regulation by peer organizations or purchasers (internal) and payment (external).

**Table 1:** Types of Incentives in Medical Practice

	Implicit	Explicit
Internal	Trust: Hippocratic oath	Regulation (e.g., by professional organizations and purchaser agencies)
External	Trust of consumers Trust of providers	Payment

Other articles in this issue focus on explicit incentives: regulation and payment. Here we argue that more emphasis should be put on implicit incentives to encourage the provision of higher value services. With respect to implicit internal incentives, we focus on mechanisms that aim to both motivate and inform all providers to improve quality. Regarding implicit external incentives, we describe accountability initiatives that help ensure that providers deliver a minimum level of quality and/or value.

## Incentive Neutrality

There are many mechanisms for paying physicians (e.g., fee‐for‐service, capitation, and salary), with some better than others (Robinson 2001). Any payment system has embedded in it strong incentives to act one way or another. A particular set of payment incentives, however, is best considered in relation to alternatives. Compared to capitation, for example, fee‐for‐service provides an incentive to deliver more services. Compared to salary, though, it may provide incentives to work harder and perhaps distinguish oneself from one's peers.

Each of these broad payment systems is associated with incentives that society may consider undesirable. It is natural, then, to try to think of ways to ameliorate these “bad” incentives. For example, one can attempt to move payment to *incentive neutrality* and then, depending on the context, make a deliberate and tempered shift from neutrality to accomplish broad policy objectives. Incentive neutrality is an idealized system in which the providers act in the best interest of the patients by providing high‐quality care and respecting their wishes, while eschewing personal gain and, at the same time, being mindful of societal interests in preserving scarce resources. This is an idealized goal as, by definition, incentives cannot be totally neutral.

Although mostly aspirational, incentive neutrality can be operationalized to some extent. Indeed, there have been efforts to modulate strong incentives embedded in base payment models to try to approach incentive neutrality, usually by blending payment approaches. For example, a recent paper about the widely admired Danish primary care system notes that for years policy makers have sought to balance capitation and fee‐for‐service incentives, and that health economists recommend this mixed payment system without agreeing on the percentages for the two components. The system tries to combine two types of incentives: (1) the treatment of patients on the [physician's] list irrespective of how often they consult the general practitioner and (2) an incentive to work efficiently when seeing patients. The challenge is to strike a good balance (Pederson, Andersen, and Sondergaard 2012, p. S35).

In insurance programs characterized by so‐called flat of the curve medicine, where more services generally do not improve outcomes, payment might be used to encourage lower spending, attempting to achieve overall incentive neutrality. But in many Medicaid programs with populations that often have lacked access to good health care, payment systems on the upward slope of the outcome curve might tilt in favor of encouraging more care.

At this time for most Americans, dominant societal, clinical, and patient‐generated influences lead physicians to do more, often too much. Such influences prominently include the following:

- Patient demands, often fostered by direct‐to‐consumer advertising and American consumer culture, which are further intensified by insurance‐induced moral hazard;
- The “technologic imperative” (Fuchs 1968), that is, the medical tradition of giving the best available care that is technically possible, the only legitimate constraint being the state of the art;
- “Defensive medicine,” driven not only by professional liability concerns but also by caring for patients whose clinical circumstances are not previously known to those providing care, for example, in emergency rooms;
- The bias in reporting research results and educational programs to emphasize positive results; and
- Direct‐to‐provider marketing by device manufacturers and pharmaceutical companies.

In this “more is better” environment, reliance on fee‐for‐service payment exacerbates the provision of too much medical care, especially if provider payments far exceed the cost of producing the services. Payment incentives could attempt to moderate the direction of these other factors by trying to influence provider behavior to resist some of these forces that commonly lead to service overuse.

## Marginal versus Embedded Incentives

It is important to distinguish between incentives that are inevitable in any payment model and incentives that derive specifically from providers’ performance against selected performance metrics. Implicit incentives *embedded* in a payment approach become part of context. They help formulate the organizational culture affecting overall behavior, but they do not include rewards and penalties for performance on specific, measureable elements of services provided. These implicit incentives therefore reduce “teaching to the test” concerns that arise with the use of financial incentives and avoid many of the difficult conceptual and technical issues both in defining valid and reliable measures and in measuring them accurately.

P4P systems rely on *marginal incentives*: increments or decrements in payment based on particular, measurable achievements. These may take away physician autonomy to treat patients in the way they believe is most appropriate. Moreover, currently researchers are unable to adequately measure (and therefore pay for) the things that are most important. Instead of measuring performance on core attributes of the provider being considered, P4P policies often rely on less salient elements of performance because of limitations in what can be accurately measured with available data sources and through provider self‐reporting. The current policy preoccupation with performance measurement has obscured the fact that changing basic payment models, without having to measure performance for specific clinical activities or conditions, may be more successful at achieving increased value than a regime of marginal financial incentives.

We argue that from a policy perspective it would be preferable to focus on getting the incentives in basic payment approaches right rather than diverting attention to marginal incentives. For example, the Medicare Physician Fee Schedule has major distortions in payment amounts in that for some services, especially tests and minor procedures, payments far exceed marginal costs of production (MedPAC, 2014b). Embedded payment rates that deviate substantially from incentive neutrality may drive physician behavior far more than the marginal incentives that are part of Medicare P4P programs. In short, how health professionals spend their time, and what additional tests and procedures they order and perform, can be a major determinant of value, even if individual clinician performance on a few clinical items is not being measured.

Furthermore, embedding incentives in improved basic payment approaches can be more efficient and reliable than measuring and rewarding performance using marginal incentives. For example, when receiving generous fees for providing erythropoietin (EPO) supplements for Medicare patients on dialysis, dialysis providers pushed hemoglobin levels too high, leading to cardiovascular complications. In response, CMS began to measure hemoglobin levels and financially penalize providers whose patients exhibited excessively high hemoglobin levels. Now, however, CMS has reduced complexity while pursuing the same objective by simply bundling the dollar value of EPO into the dialysis payment bundle, eliminating the prior incentive to overuse EPO in the first place (MedPAC, 2014a).

## Specific Concerns about Using Financial Incentives That Rely on Performance Measurement

Although P4P and public reporting both rely on performance measurement, and often the same measures, their purposes differ. While P4P provides a direct financial incentive to providers to meet the external tests of performance, public reporting has several purposes. First, it provides information to consumers so they can make more prudent provider choices, which then affect market share; poorer performers based on the publicly reported measures are penalized financially unless they improve. Indeed, if realized, this objective of public reporting could be an even more powerful financial incentive than P4P, which is usually limited to a few percentage points of payment up or down. However, as reviewed by Roland and Dudley in this volume, consumers generally do not use such information in their selection of providers. As a result, provider revenues have not been materially affected.

Second, public reporting permits health professionals and provider organizations to see how their own performance compares to peers. In that way, it supports nonfinancially based attributes such as pride, concern about reputation, and desire for self‐improvement grounded in professional duty to act in patients’ best interests, thus encouraging intrinsic motivation. However, because public reporting affects reputation, it places a higher burden on the meaningfulness and accuracy of performance measures themselves. These measures must be indisputably important reflections of the quality of the provider being measured and also be statistically valid. Policy makers tend to ignore those requirements with current and impending public reporting regimes, such as Medicare ratings of physicians, in which the available measures often miss the essence of what many physicians in diverse specialties are expected to do for patients (Berenson and Kaye 2013).

The article by Roland and Dudley reviews the theory and empirical findings on the strengths and weaknesses of performance measurement as part of public reporting and P4P programs. However, we have three areas of concern that such approaches may inadvertently compromise professionalism. They replace altruism and intrinsic motivation with pecuniary incentives; employ easier‐to‐measure, but not necessarily the most appropriate metrics of quality; and require appropriate, objective data, which is problematic.

### Replace Altruism and Intrinsic Motivation with Pecuniary Incentives

Money clearly matters, and it would be difficult to argue with the notion that money is a key objective to a for‐profit, publicly traded organization. Nevertheless, evidence suggests both that individuals choose professions, and organizations choose to behave, for reasons not explained by financial rewards. Physicians provide a good example. A review of 41 articles published about medical training in the United States between 1986 and 2006 emphasized the key role of values in career choices, including “the values of the individual, the values of the educational organizations in which individuals train, and the values of groups to which individuals belong” (Borges et al. 2010). Similar motivations appear to underlie career choices outside of the United States (Crossley and Mubarik 2002; McManus, Livingston, and Katona 2006). Commonly noted motivations include helping patients, engaging in fulfilling and interesting work, autonomy, self‐respect, professionalism, and reputation.

One essential difference between health care and many others parts of the economy is the preponderance of nonprofit organizations, such as community and academic hospitals. While such firms do need to meet a bottom line—“no money, no mission”—they generally are driven by other motivations, including the following:

- Meeting their mission and requirements of preserving their not‐for‐profit status, which includes elements of serving the community at large by providing access to quality health care services and treating their employees well
- Garnering a good reputation that reflects well on the trustees (if any), administrators, and medical staff

If people are motivated by altruism or nonpecuniary intrinsic factors such as doing a good job, and organizations have a broader goal than maximizing profits, then financial incentives might not be the most effective way to influence their actions. In the health arena, a commonly cited example is blood donations.

In professions that involve the highest levels of independence and judgment, personal motivation and self‐esteem are critical. If physicians adopt an attitude of “you get what you pay for” then patients and society are in jeopardy of losing a great deal. In this regard, Martin Roland expressed concern that under P4P you could “[end] up with a system where, essentially, doctors only did anything because they were paid for it and had lost their professional ethos” (Galvin 2006). This is not meant to be a blanket indictment of financial incentives in health care. More nuanced approaches, such as rewarding organizations and teams within organizations rather than individuals, may remove some (but not all) of the potentially deleterious manifestations of P4P (Wynia, 2009; Berenson, Pronovost, and Krumholz 2013).

### Employ Easier‐to‐Measure, but Not Necessarily the Most Appropriate, Metrics of Quality

“If the only tool you have is a hammer, [it is tempting] to treat everything as if it were a nail” (Maslow 1966). The P4P hammer provides rewards and penalties if providers do—or do not do—very particular things. CMS's sponsored Premier Hospital Quality Incentive Demonstration Project (HQI) provided hospitals clear incentives to perform in the top tier on particular measures, which evidence suggests should be performed on patients with coronary care, pneumonia, and hip and knee replacement.

Hospitals in the HQI study have a clear incentive to focus on such measures. But would fulfilling the measures actually improve quality outcomes for these conditions? A qualitative study (Curry et al. 2011) suggests not always. It involved interviews with hospital personnel focused on six domains: hospital practice and protocols for improving acute myocardial infarction (AMI) care, organizational values and goals, senior management involvement, broad staff presence and expertise in AMI care, communication and coordination among groups, and problem solving and learning. The findings were striking: while the high‐ versus low‐performing hospitals differed markedly on the last five factors, there were no differences in practices and protocols for improving AMI care. The authors conclude that several organizational features might reduce risk for death within 30 days of admission for patients with AMI. First, having clear values and goals to be the best, coupled with the strong engagement from staff members of diverse disciplines, senior management, and staff, focuses attention and resources on the issue of quality of care. Second, medical errors and preventable deaths occur in part because of poor communication or “dropping the ball” during transitions of care, which suggests that strong communication and coordination among groups probably limit errors in transitions and enable a more reliable and safe environment at a hospital. Finally, solving problems in a way that seeks and addresses root causes, a practice that was endemic in the top‐performing hospitals, may ensure that difficulties in processes are addressed swiftly and routinely and reduce the risk inherent in the hospitalization and complex clinical care of patients with AMI (Curry et al. 2011). In short, while it should be fairly straightforward to ensure that particular pills are dispensed at particular times, it is exceedingly difficult to instill values in hospital staff, improve communication and coordination of care, and find the root causes of lagging performance. The concern here about P4P and public reporting programs, then, is that they may focus on the relatively easy‐to‐measure items, but leave unmeasured and unconsidered the more important determinants of quality, not only for the particular conditions being looked at but for overall care. Improvement on these more fundamental issues entails a much greater human commitment and resource expenditure but has little role in current use of performance measures. Quality of care would be fostered with a much stronger emphasis on the outcomes of care rather than the current emphasis on process measures. Measuring important health outcomes is a formidable challenge and still a work in progress, though worth the effort (Berenson, Pronovost, and Krumholz 2013).

### Require Appropriate, Objective Data

Measurement of provider performance is essential for compiling public health care quality report cards and for implementing P4P programs. One problem is that performance measures rely to some extent on self‐reported data. While the organizations compiling the data engage in a certain amount of auditing, it is hardly universal. If a hospital or doctor is paid for engaging in certain care processes, they are more likely to *report* that they did so in the medical records. Similarly, if they are penalized when something adverse occurs, then they are less likely to report such events.

The problem of providers misreporting data for public consumption, such as on their websites, has been discussed for some time (Pronovost, Miller, and Wachter 2007). The issue here is whether providers can influence the payment they receive by providing inaccurate or misleading data to payers. When CMS stopped paying for some adverse events—for example, leaving a foreign object in the body during surgery or central line‐associated bloodstream infections, recording of these adverse events declined by 50 percent in just a quarter. Other clinical data, however, showed no actual reduction (Farmer, Black, and Bonow 2013). Similarly, the *New York Times* (Thomas 2014) reported how nursing homes that provided poor quality care were receiving five‐star ratings from Medicare, because the ratings are based in large part on self‐reported data by the nursing homes that the government does not verify. In response, CMS announced that it was revising its rating system to enhance verification of the data.

Advocates of public reporting and P4P often acknowledge that current measure sets have major gaps, while accurate measurement faces statistical validity challenges, especially when attempting to assess individual clinician, rather than organization, performance. However, they often proceed to argue that one needs to start somewhere—that if measurement actually becomes an integral component of payment policy, momentum for significant improvement in both measures and measurement would inevitably follow. In the recently enacted Medicare Access and CHIP Reauthorization Act of 2015 (MACRA, 2015), the legislation that repealed the Sustainable Growth Rate constraint on the Medicare Physician Fee Schedule, Congress decided to significantly raise the financial stakes of P4P in what Congress labeled the Merit‐Based Incentive Payment System. They accomplished this by moving to penalties and rewards for individual physician performance of 9 percent, that is, as much as an 18 percent potential swing in payment based on performance on a handful of measures. This is despite the fact that large gaps in available measures remain unfilled and likely will for the foreseeable future.

In contrast, we believe that weak, often irrelevant measures and invalid measurement assessments can breed cynicism among clinicians, further compromising already threatened intrinsic motivation that professionals have to act in the best interests of their patients and society. Even performance measure advocates are aware of the often counterproductive impact of the growing reliance on measures, noting that some measures may help improve care, while others at times enrage colleagues or prompt expenditures that produce no care improvements (Cassel et al. 2014).

## Limited Role of Financial Incentives in Quality Improvement

Unfortunately, there is worrisome evidence in health care that financial incentives based on performance measures can seriously undermine professionalism and commitment to patient well‐being and may not necessarily improve patient outcomes. For example, a recent study found a surprising spike in postoperative cardiac surgery deaths at day 31, when physicians were being measured on their 30‐day mortality rates. The clear implication was that end‐of‐life decision making, requiring withdrawal of aggressive treatment, was postponed beyond the 30‐day measurement period (Maxwell et al. 2014). This study adds to the literature finding that physicians avoid intervening in critically ill patients because of concerns about how they will fare under public reporting of their performance (Narins et al. 2005; McCabe et al. 2013).

Earlier this year, the *Journal of the American Medical Association* published two original research papers showing that participation in a surgical outcomes reporting system—the American College of Surgeons’ National Surgical Quality Improvement Program (NSQIP)—was not associated with improvements in clinical outcomes (Etzioni et al. 2015; Osborne et al. 2015). A companion paper that also relied on NSQIP data, rather than administrative data, found that readmissions after surgery were mostly attributable to surgical complications rather than to issues related to deficient transitions from the hospital to the ambulatory setting, as is common with medical admission (Merkow et al. 2015).

Commenting on these findings, two highly respected physician quality experts contributed important insights about their implications (Berwick 2015; Leape 2015). Citing the remarkable success achieved by the Michigan Keystone Project at eliminating central line–associated blood stream infections in Michigan hospitals, Leape (2015) emphasized that the most powerful methods for reducing medical harm are feedback, learning from the best, and working in collaboration—all approaches that support intrinsic motivation. He also pointed out that NSQIP members have a “priceless asset: comparative data,” not publicly reported but provided to all the participant organizations in the collaboration. Importantly, the data are used to support intrinsic motivation to foster quality improvement rather than an explicit economic incentive to perform in a particular manner. Providing support for the importance of intrinsic motivation, Kolstad (2013) recently found that publicly reported quality measures improve performance by altering surgeons’ beliefs about their own quality relative to a reference set of peers, rather than by altering consumer demand and moving market share.

Berwick (2015) explained that the failure of NSQIP hospitals to perform better than nonparticipant hospitals obscured the fact that all hospitals in the study reduced mortality rates, serious complications, and readmissions by comparable amounts. He suggested that many hospitals seem to be embedding quality improvement “in their DNA,” a view consistent with the finding by Curry et al. (2011) on the central role of hard‐to‐measure institutional culture and values in achieving better outcomes.

Recently, the New York Times reported on “A Sea Change in Treating Heart Attacks,” citing data from the American Heart Association showing that the death rate from coronary heart disease fell about 38 percent from 2003 to 2013. This drop was largely because hospitals had developed practical work process improvements to slash the time it takes to clear blockage in a patient's arteries to get blood flowing again to the heart muscle (Kolata 2015). The article emphasized that this dramatic quality improvement occurred in the absence of new medical discoveries, new technologies, or new payment incentives, and with little public notice. What instead was largely responsible was a national campaign directed by the American College of Cardiology and the American Heart Association, working with physicians, paramedics, and other health professionals and with hospital staff to “adopt common sense steps” to improve performance. They especially targeted the time it takes the hospital to transfer patients from arrival at the hospital to the catheterization lab to clear the blockage and prevent heart muscle damage (Cannon et al. 2000; Antman et al. 2004; McNamara et al. 2006). The story recounts that CMS had created a national database showing how long it took hospitals from patients’ arrival to open their blocked arteries. Furthermore, the top performers on this measure served as models that resulted in development of work process protocols that all hospitals could follow to streamline the process of getting patients to the catheterization lab.

CMS's Hospital Compare Program did publicly report hospital performance, suggesting that careful public reporting of widely accepted, important measures of performance that directly affect outcomes can positively support intrinsic motivation (Khare et al. 2010). In this case, the affected physicians and hospitals clearly respected the value of measures on which they were being assessed.

In short, financial incentives are not central to the successes described in these reports. Data are crucially important to support quality improvement activities, but not necessarily with public reporting and certainly not to support P4P schemes. Rather, the successes seem related more to fostering a supportive culture relying on professionals’ intrinsic motivation and organizations’ mission to improve care, accompanied by straightforward quality improvement methods to produce actionable, common sense steps. Furthermore, the growing evidence base finds little support for the presumption that public reporting and P4P programs, above and beyond the use of data to support quality improvement activities, actually improve processes of care or patient outcomes (Chatterjee and Joynt 2014).

## Non‐financially Based Initiatives to Improve Quality and Accountability

Public policy can be fashioned to better support a quality improvement culture that goes beyond the current preoccupation with crafting marginal financial incentives to support and facilitate intrinsic motivation to improve care quality and accountability. Examples include CMS's Health Care Innovation Awards (HCIA) Partnership for Patients Program designed to improve population health (Kassler, Tomoyasu, and Conway 2015), and Conditions of Participation in Medicare and Medicaid. These and other CMS activities tend to be marginalized in public policy discussions and budget allocations because of the disproportionate emphasis on financial incentives as the dominant policy response to ongoing quality and safety problems in U.S. health care generally and Medicare specifically (Blumenthal, Davis, and Guterman 2015; Rajkumar, Press, and Conway 2015).

Commenting on the difficulties inherent in evaluating a program like Partnership for Patients, which involves most of the hospitals in the country (making a control group of hospitals needed for a formal evaluation virtually impossible), Casalino and Bishop (2015) argued that it is particularly difficult to evaluate the impact of CMS's broad, collaborative quality improvement programs. The converse is that it is much more feasible to rigorously evaluate the Innovation Center's payment incentive programs. Just as serious difficult‐to‐measure quality problems, such as diagnosis errors, tend to get marginalized in public policy, innovations not amenable to relatively easy evaluation tend to be given short shrift as well.

Little is known about the effectiveness of the various value‐enhancing approaches that do not involve new payment models, public reporting or P4P. Yet they have been well funded by CMS in recent years if not much discussed. Some wonder whether these programs represent money well spent or simply a “feel good approach” without a positive payoff (McKinney 2014; Pronovost and Jha 2014). We assert that, despite lack of evidence for effectiveness or ineffectiveness, that many promising and long‐standing programs have the potential to pay off in many cases.

One example is value‐based purchasing, the term adopted in Medicare in place of “pay‐for‐performance,” but which is in fact a much broader approach to achieving higher value than possible just through public reporting of measures and marginal financial rewards and penalties. Value‐based purchasing attempts to address both accountability aimed at assuring an acceptable floor of minimally acceptable care, and quality improvement aimed at broadly raising the quality of acceptable performance. The role of purchasers in influencing provider quality and efficiency of care has shifted over the past two decades, from “cutting off the tail” of substandard quality to “raising the mean quality” for all. This is based on the view that raising the mean would be more willingly accepted by providers and would have greater total impact on patient well‐being than even successful efforts to reduce substandard care. This was epitomized in the shift in the orientation and accompanying name change of Professional Review Organizations to Quality Improvement Organizations in 2001 (Bradley et al. 2005).

Yet it can be argued that the higher priority for accountability should be to protect against substandard care delivery. That means focusing on the easier‐to‐identify tail of the quality distribution than the care in the broad reasonable average. For improving the average, we believe that government's role should be more in facilitation, encouragement, and assistance to take advantage of health professionals’ and other providers’ intrinsic motivation to improve the care they provide.

## Quality Improvement

We believe that a key approach to quality improvement is fostering, perhaps even demanding, local responsibility for quality improvement, but not imposing the precise approach and measures that the local actors have to use. We also appreciate the quality improvement benefit of a strategic use of measures as in CMS's Partnership for Patients, internally used targets of progress as in the Medicare Conditions of Participation, and individual organizational procedures to promote evidence‐based medicine in accountable care organizations (ACOs).

Clinicians and other health professionals likely respond much more positively, with a greater sense of cooperation, to performance measures that they had a hand in developing than when imposed from above. The problems associated with imposing quality measures and other syntheses of “evidence” can be seen with physicians’ responses to the Physician Quality Reporting System (PQRS) measures, which represent the core of Medicare's physician value‐based purchasing. As of 2013, 6 years from inception, only half of 1.25 million eligible health professionals were participating in PQRS (CMS, 2015), suggesting both excessive reporting burden and lack of clinician respect for the worthiness of the measures and the value of reporting (Berenson and Kaye 2013). Because Congress, through MACRA, significantly increases the rewards and penalties for P4P in the new Merit‐Based Incentive Payment System, more physicians will feel the financial pressure to participate even if there is no improvement in the measures. Alternatively, physicians will feel financial pressure to participate under an Alternative Payment Model, such as those paying accountable care organizations and bundled payments, to receive 5 percent higher payment, irrespective of whether they have a commitment to practicing differently under the new models. Many will feel somewhat coerced to participate, not an auspicious start of delivery system and payment reform efforts.

Our approach to quality improvement is captured by the title “A New Strategy to Improve Quality: Rewarding Actions Rather Than Measures” (Werner and McNutt 2009), which emphasizes local responsibility for QI. Among professional groups, internally driven efforts that function as communities of learning and change social norms are highly effective tools to improve performance, but are not well developed in health care. The approach has been dubbed “communitarian regulation” (Pronovost and Hudson 2012).

Berenson, Pronovost, and Krumholz (2013) call for a more strategic use of measures by Medicare and other payers to address important problems. An apparently successful approach to strategic use of measures to support a collaborative approach is CMS's Partnership for Patients. A recent AHRQ analysis showed that approximately 1.3 million fewer patients were harmed in U.S. hospitals between 2010 and 2013. This represents a cumulative 17 percent reduction or prevention of about 50,000 deaths, which resulted from a reduction of hospital‐acquired conditions (HACs). Although many believe that financial incentives have driven these reductions in HACs, in fact, the financial incentives that apply to hospitals resulted in only small changes in improving quality before the initiation of Partnership for Patients (McNair, Luft, and Bindman 2009; Lee et al. 2012; Meddings et al. 2012; Waters et al. 2015). It is plausible that the estimated 3‐year cost savings of nearly $12 billion (AHRQ, 2014) was attributable in great part to the collaborative approach adopted in Partnership for Patients, with financial incentives playing an ancillary role.

An important corollary to using measures to support quality improvement is that when an important quality problem does not lend itself to accurate measurement, it nevertheless can be addressed with other strategies identified in broadly conceived value‐based purchasing, such as through collaboration, feedback, and technical assistance. An example of such a quality problem is that of diagnosis errors, a major, if largely ignored problem, for which accurate measurement is particularly difficult (Singh and Sittig 2015).

Public policy could place expectations on provider organizations to engage in substantive quality improvement activities and to demonstrate actual work being done, with providers producing internally used targets of progress and carrying out remedial action when targets are missed. Medicare Conditions of Participation (CoPs) offer an example of the approach, but without the needed evaluation to assess the success of the approach.

For the Medicare Shared Savings Program that establishes ACOs, the regulations require the ACOs to have in place their own procedures and processes to promote evidence‐based medicine, beneficiary engagement, and coordination of care (Federal Register, 2015). However, CMS apparently did not review and comment on an ACO's specific approach and did not follow up to examine progress against an ACO's own benchmarks. Nor did CMS adopt a strategy of seeking to disseminate innovative approaches to addressing these and other important domains of patient care, which are often not amenable to accurate measurement. Instead, performance measurement has been the preoccupation for both quality improvement and accountability, requiring ACOs to develop detailed action plans to improve performance on individual quality measures, even as ACOs themselves and the MedPAC criticize the measurement burden and the usefulness of the measures in use. (Evans 2013; MedPAC, 2014c).

## Related Quality Improvement Opportunities

A broad commitment to local quality improvement should produce a range of innovative approaches that can become best practices and be disseminated. We describe four approaches to empowering, or at least facilitating, robust local quality improvement, some of which can be supported at a national level. These include promoting quality improvement collaboratives among providers; developing partnerships among public and private payers and providers; follow‐up and feedback from other providers and patients; and recognition and support of local quality improvement projects.

### Promoting Quality Improvement Collaboratives among Providers

A number of organizations have promoted models of engaging health professionals and provider organizations in collaboratives across institutions and geographic areas. Examples include Regional Health Improvement Collaboratives, the Institute for Healthcare Improvement (IHI), and the Premier Hospital Alliance. These efforts have resulted in broad interest and some clinician participation, with some evidence of successful improvement in outcomes. However, to date these initiatives have mainly attracted so‐called first movers and early adopters.

The Network for Regional Healthcare Improvement is a national organization representing over 30 member organizations working in their regions, while collaborating across regions, to support the triple aim of improving the health of populations, reducing per capita spending, and improving the patient experience of care. These are “bottom‐up” efforts. Although often centered on provider activities, they are multistakeholder organizations with involvement of payers, purchasers, and consumers (NHRI, 2014). The regional collaborative approach is based on the recognition that approaches to improve care will differ based on the varying characteristics and health care cultures of different communities (ROOTS, 2011). These programs emphasize identifying and disseminating best practices for particular conditions, and provide training and coaching to practitioners on ways to analyze and implement improvements in health care delivery (NHRI, 2014).

There has not been a formal evaluation of the outcomes associated with collaboratives, but there have been impressive success stories. Examples include the regional collaboratives improvement in outcomes for diabetics in Cincinnati (RWJF, 2013), reduction in hypertension in Wisconsin (WCHQ, 2014), and integration of behavioral health with primary care in Pittsburgh (PRHI, 2014). Similarly, IHI reports that its 100,000 Lives Campaign, involving a collaboration of over 3,000 hospitals, has saved over 120,000 lives (IHI, 2006), although there are grounds for some skepticism about the magnitude of the self‐reported, lives‐saved estimate (Berwick, Hackbarth, and McCannon 2006; Wachter and Pronovost 2006). Much of the progress in such collaboratives seems to lie with organizational leadership and some dedicated clinicians and others rather than the typical, busy practitioner. However, there have been successes involving practicing clinician participation with emphasis on group learning and mutual support.

Birkmeyer and Birkmeyer (2006), observing the paucity of measures for assessing the quality of care performance of surgeons, proposed “pay‐for‐participation” in collaboratives as a more useful and relevant alternative to pay‐for‐performance, at least for surgeons. It might be hard to justify paying extra for such participation, given surgeons’ already relatively high incomes. Active and meaningful participation in quality‐enhancing collaboratives might provide credit for health professionals in a comprehensive, value‐based purchasing regime that CMS would administer. However, effective protections against token participation compliance would need to be developed. Instead of reducing fee schedule payment rates and then using this pool to pay those who qualify based on their performance on particular performance metrics, as enacted in MACRA, physicians might achieve their bonuses through meaningful participation in collaboratives of various kinds. These may include programs sponsored by physician specialty societies, such as those described earlier that helped produce a major drop in heart attack deaths.

### Developing Partnerships among Public and Private Payers and Providers

One related, collaborative model in which CMS was the lead convener is Partnership for Patients. According to CMS, the program is a public–private partnership that seeks national change by setting clear aims, aligning, and engaging multiple Federal partners and programs, private partners and payers, and establishing a national learning network through a CMS investment in 26 hospital engagement network (HEN) contractors (AHRQ, 2014). The HENs successfully enrolled more than 3,700 acute care hospitals in the initiative, accounting for 80 percent of the nation's inpatient discharges, and had these hospitals engaged in achieving the aims throughout 2012, 2013, and 2014.

### Follow‐Up and Feedback from Other Providers and Patients

Some quality and safety breakdowns are memorable, if not measureable. As we have emphasized, one useful purpose of performance measurement is to provide data that physicians and health care organizations can use to compare their own performance to those of peers. Schiff (2008) has advocated a systematic approach to providing feedback to practicing clinicians on diagnosis outcomes and error, for example, emphasizing an approach that “fully involves patients and possesses an infrastructure that is hard wired to capture and learn from patient outcomes.” Related research has demonstrated that physicians readily recalled multiple cases of diagnostic errors and were willing to share their experiences for quality improvement purposes (Schiff et al. 2009). Potentially, public policy could encourage systematic feedback to address a range of quality and safety problems, although the effectiveness and feasibility of the approach would need to be established. Systematic feedback about errors, patient‐reported outcomes, and other aspects of care would also provide an important role for consumers and patients (Schiff 2008; PROMIS, 2015).

The Medicare Improvements for Patients and Providers Act of 2008 (MIPPA) directed the Department of Health and Human Services to develop a program to give physicians confidential feedback on the resources used to provide care to Medicare beneficiaries and performance on some quality measures. In response, CMS has established and implemented the Physician Feedback Program by distributing feedback reports to an increasing number of physicians providing data on resources used and quality. MIPPA also mandated that GAO (Government Accountability Office [GAO] 2011) conduct a study of this program, which found that CMS faces challenges incorporating resource use and quality measures for physician feedback reports that are “meaningful, actionable, and reliable.” Unfortunately, such challenges to measurement seem to be a recurring theme in the health care measurement literature.

### Recognition and Support of Local Quality Improvement Projects

Starting in May 2012, CMS has supported Health Care Innovation Awards, providing more than $1.25 billion to organizations that are implementing the most compelling new ideas to deliver better health, improved care, and lower costs to people enrolled in Medicare, Medicaid, and the Children's Health Insurance Program (CHIP), particularly those with the highest health care needs. Innovative projects worthy of substantial financial support are expected also to provide Medicare, Medicaid, and CHIP savings that exceed their costs (CMS, 2014a). CMS has funded awards to providers, payers, local government, public–private partnerships, and multipayer collaboratives. Each grantee project will be monitored for measurable improvements in quality of care and savings generated, presumably with a goal of exporting lessons learned for broader application. Grantees will also be subject to independent evaluation by CMS contractors. Through two rounds of funding, CMS has funded over 146 recipients spanning all states (CMS, 2012a, 2014a). Grants include a broad range of interventions, including a new approach to supporting patients experiencing first episodes of psychosis, applying telemedicine approaches for patients needing critical care expertise in rural areas, and greater reliance on paramedics to visit patients in their homes (CMS, 2012b, 2014b).

In addition to this underappreciated approach to promoting innovation, greater effort might be extended to identify best practices with accompanying awards for innovative and effective QI projects. Although some organizations, such as IHI, already disseminate best practice lessons based on local initiatives, greater transparency of these specific projects, with attendant removal of identifying patient and provider information, would provide many more QI models worthy of adaptation in other provider environments. In addition, to promote excellence and initiative, a public and private collaboration could recognize and reward meritorious projects modeled after the Malcolm Baldrige National Quality Award, the national quality award that recognizes U.S. organizations in the business, health care, education, and nonprofit sectors for performance excellence (Malcolm Baldrige National Quality Award, 2014).

## Accountability Mechanisms

### Conditions of Participation

The core accountability mechanism in Medicare is the requirement that most institutional providers and suppliers must meet CoPs—the programmatic standards that have to be met to be allowed to participate in the Medicare and Medicaid programs (Federal Register, 2012). They are entry requirements. Although some of the standards specify a quality improvement program, this approach basically establishes baseline expectations—standards—for participation, largely to assure basic protection of beneficiaries. This approach does not include providing differential payment based on performance against measures. CMS directly inspects providers through a survey and certification process or, alternatively, deems (i.e, accepts the determination of) accrediting organizations, such as the Joint Commission, which are able to demonstrate that their health and safety standards and survey and oversight processes meet or exceed those used by CMS.

Although an essential part of the accountability framework in Medicare and Medicaid, CoPs have received little attention in public policy. The emphasis on public reporting of quality performance has taken center stage, even as periodic changes are made to the standards through public rule‐making. The Institute of Medicine reviewed the state of hospital CoPs in a 1990 Report: A Strategy for Quality Assurance in Medicare (IOM, 1990). Little policy analysis of the role and operations of CoPs has been forthcoming since.

Although we know very little about how effective this approach is, there is nevertheless reason to believe CMS is not adequately funded to carry out its oversight responsibilities. The *Washington Post* recently published a front page story, “Selecting Hospice Is a Roll of the Dice for Families.” It emphasized that quality and safety of hospices vary tremendously and that patients and their families lack useful quality measures to help in their selection (Whoriskey and Keating 2014). Government inspections of hospices apparently typically take place only every 6 years (OIG 2013). Yet for years the National Hospice and Palliative Care Organization, a hospice trade association, has supported the need for more frequent surveys of hospice providers to better assure basic patient protections in hospice and to directly address the kind of abuses documented in the Post article and elsewhere (National Hospice and Palliative Care Organization [NHPCO] 2014). Congress recently passed the Medicare Post‐Acute Care Transformation Act (IMPACT Act), which, among other things, mandates that all Medicare‐certified hospices be surveyed every 3 years for at least the next 10 years (National Hospice and Palliative Care Organization [NHPCO] 2014).

The concept of participation requirements to assure basic accountability could also be extended from facilities to physicians, as done in other countries. For example, in the Danish system, to receive payments, general practitioner offices are contracted to be open on four weekdays from 8:00 AM to 4:00 PM, with the first hour reserved for telephone calls with patients. On one weekday, hours need to run to 6 or 7 PM (Pederson, Andersen, and Sondergaard 2012). In short, the Danish contract serves as the equivalent of CoPs, with the focus on assuring basic levels of services provision. A similar concept could be adopted in Medicare.

For physicians in particular, policy makers might consider using board certification as a condition of participation. However, although studies show that board‐certified physicians provide statistically significant higher quality care than those without certification, the quality differences were relatively small. Given other important considerations, such as patient access and the benefits of competition, it would be hard to justify a requirement for board certification as a condition of participation for physicians (Sharp et al. 2002; Chen et al. 2006; Reid et al. 2010). Furthermore, initial findings do not demonstrate that periodic Maintenance of Certification is associated with higher quality, although spending was reduced (Gray et al. 2014; Hayes et al. 2014).

### Termination of Providers with Evidence of Unacceptable Performance

As emphasized earlier, it is much more difficult with available performance measures to distinguish among providers with acceptable if variable performance than it is to identify substandard or unacceptable performance. To date, CMS has not acted assertively even when profiles of claims data reveal billing patterns that deserve sanction, if not termination, from Medicare participation. For example, Pro Publica has reported that physicians with unusual Medicare billing patterns often have been disciplined not by Medicare, but rather by their state medical licensing boards or have faced accusations against their licenses. They have also found dozens of physicians who Medicare kept paying as acceptable providers after they were suspended or terminated from state Medicaid programs, indicted or charged with fraud, or had settled civil allegations of submitting false claims to Medicare (Ornstein 2014).

Although the example provided raises the issue of why CMS seems to tolerate billing fraud, there is no reason why it could not take more proactive steps to identify substandard clinical performance by mining claims data to identify outliers on a range of clinical issues, such as rates of admissions for ambulatory care‐sensitive conditions, rates of particular elective procedures, and/or readmission rates after surgery, as screens to identify potential, substandard performance. Then, as with any fraud investigation, the actual clinical records would be examined before drawing conclusions about individual or group culpability, following a due process approach to fact‐finding and judgment. Today, CMS and its administrative contractors have the authority to take actions against health professionals providing substandard quality to beneficiaries. However, the recent focus on quality improvement to raise mean performance, along with the scarce resources available to engage in detailed clinical review, have inevitably limited CMS's actions in this area (Eichenwald, 2003; Eisler and Hansen 2013).

### Targeted Prior Authorization

In 2008, the GAO recommended that CMS examine the feasibility of requiring prior authorization for imaging (Government Accountability Office [GAO] 2009). In 2011, MedPAC suggested an approach to require documented high‐use practitioners to participate in a prior authorization program for advanced imaging, that is, MRI, CT, and PET studies (MedPAC, 2011). A core part of the program would be the targeting of (case mix adjusted) physician outliers. This permits targeting of scarce administrative resources, while avoiding the imposition of new burdens on most clinicians. In 2014, Congress adopted a form of such a prior authorization program for particular imaging services, when ordered by health professionals considered outliers. They relied on appropriate use criteria developed or endorsed by professional specialty societies and administered by CMS. The program is scheduled to commence in 2020 (MACRA, 2015).

Beyond imaging services, prior authorization could be adopted more broadly by Medicare, relying on lessons learned over the years that the approach works best for high‐priced, elective interventions where there is demonstrated practice variation and reasonably strong evidence about appropriateness (Berenson, 2003). As private insurers do, Medicare could incorporate prior authorization into national and local coverage determinations where appropriate, rather than relying, as now, on largely ineffective and inefficient “pay‐and‐chase”—after the fact (Tunis et al. 2011).

## Conclusion

While policy makers pay increased attention to formulating ways to improve health care outcomes in the most efficient manner, this attention is largely and increasingly focused on methods designed to increase providers’ extrinsic motivation. Two of the main methods are pay‐for‐performance and public reporting of performance. We argue that this narrow focus will impede our search for improved performance, efficiency, and quality.

The evidence provided in this article indicates that efforts to “fine tune” provider behavior through financial incentives aimed at influencing payments at the margin show results that are spotty at best. On the face of it, this is not surprising. Such incentives focus almost entirely on pecuniary motivations. But health professionals, in particular, have a myriad of motivations beyond financial success and professional stature, perhaps the main one being helping their patients and populations maintain their health. Other incentives include mastery of an extraordinarily challenging craft. There is much satisfaction gained in doing one's job well, particularly when so much is at stake. Providing clinicians with the wherewithal to do so, including providing them with feedback on their performance that need not necessarily be shared with the public, has been repeatedly shown to be an effective way to improve care process and outcomes. Rather than having one provider pitted against another to distribute financial rewards and penalties, provision of technical assistance and encouraging quality‐related collaborations can lead to more‐desired results.

Of course, financial incentives matter, especially when they distort behavior for the worse. What we should seek is incentive neutrality, where providers deliver what is best for the patient without skewing their behavior toward personal gain, while acting in a way consistent with societal interests in avoiding wasteful care. To this end, we applaud the CMS's efforts as it works to move toward alterative payment models that reward quality, collaboration among providers, and coordination of patient care through such mechanisms as accountable care organizations, medical homes, and bundled payment (Burwell 2015).

As these efforts develop, however, it is critical to embrace a broader view of what motivates providers. It is worth remembering Martin Roland's statement, noted earlier, that measuring and reporting performance as a means of fine‐tuning payments could lead to a state of affairs where physicians are no longer focused on their professional ethos, but instead simply respond to money. Even the most ardent advocate of financial incentives would find this distasteful. Future policy initiatives will benefit from a renewed emphasis on alternative means of motivation, several of which have been presented here.

## Supporting information

Appendix SA1: Author Matrix.
